# Paediatric acute liver failure: Confirm the outbreak, find the cause and explore the mechanisms

**DOI:** 10.1002/ueg2.12306

**Published:** 2022-09-12

**Authors:** Audrey Coilly, Didier Samuel

**Affiliations:** ^1^ AP‐HP Hôpital Paul Brousse Centre Hépato‐Biliaire Villejuif France; ^2^ University Paris‐Saclay UMR‐S 1193 Villejuif France; ^3^ Inserm Unité 1193 Villejuif France; ^4^ Hepatinov Villejuif France

The recent outbreak of cases of paediatric liver failure raises numerous questions regarding the potential causal agent. Paediatric liver failure is a rare disease.^1^ Some causes are specific to children: Metabolic disorders such as Type 1 tyrosinemia, mitochondrial cytopathies, urea cycle disorders, galactosemia, hereditary fructose intolerance; genetic diseases such as neonatal haemochromatosis. Autoimmune hepatitis, acute leukemia, hypoxic shock, heatstroke, paracetamol overdose, fulminant Wilson's disease and Reyes syndrome are common cause of acute liver failure (ALF) in infants and adults. Fulminant viral hepatitis with hepatotropic viruses such as HBV, HAV, are also common in adults. Other viruses such as HSV1, HHV6 can lead less frequently to Fulminant viral hepatitis both in adults and infants. Parvovirus B19 has been associated with fulminant liver failure and pancytopenia mainly in infants.^2^ Unfortunately, in a percentage of 15%–50% of patients with fulminant liver failure, the cause remains unknown. In addition, paediatric ALF of unexplained causes have been followed, in some cases, by aplastic anaemia.^3^ In adults, despite extensive search, 20% of ALF cases remains of indeterminate origin.^4^, ^5^ The mechanisms of ALF due to hepatotropic viruses such as HBV or HAV is mainly due to the immune and inflammatory reactions against the infected hepatocytes. CMV, EBV, HHV6 are responsible of rare cases of acute hepatitis, however the evolution to ALF is exceptional. Their mechanism of action is not really known. HSV1 or 2 are probably directly cytopathic. In contrast, the role of Parvovirus and HHV6 in ALF is unclear and their role in the occurrence of ALF has been challenged. Recently an outbreak of severe acute hepatitis in children below 10 years with ALF has been reported. Data have been concentrated on children below 10–16 years according to countries, with AST or ALT over 500IU/l, an unknown aetiology of hepatitis and onset after October 2021.^6^, ^7^, ^8^, ^9^ Epidemiological data suggested an increase in the frequency of severe acute hepatitis in Children. The investigations have been negative for all main cause knowns, but in 45%–90% of cases, adenoviruses have been detected in blood, respiratory tract and stools. Current and Past Infection with SARS Cov2 have been found in 10% and 26% of cases, respectively in a US Survey.^7^ In contrast the percentage of patients who were vaccinated against SARS COV2 was very low, excluding the role of the vaccine. In the current report, five young patients developed liver failure during a course of acute hepatitis,^10^ four were aged between 11 months and 3 years, and one was 8 year‐old. ALT was above 2000 IU/l in all cases, SARS COV 2 virus was present by PCR in two patients, two others have past SARS COV 2 infection, four had Adenovirus infection present in the blood or in the stools. In two patients, adenovirus, was associated with rotavirus in one and VZV (primary infection) in another. Four children went to emergent liver transplantation and one survived spontaneously. The histological analysis of the explant liver showed massive necrosis in two cases with no inflammation, spotty necrosis with some inflammatory cells in another, inflammatory and ductular reaction in another. There were no typical features of viral hepatitis, no viral inclusions, no features of autoimmune hepatitis and finally no common histological features.

Are we facing here a new disease? First, we need additional epidemiological studies to confirm a true outbreak. Second, the presence in these five cases and in all cases reported so far of an adenovirus and a current or past infection of SARS COV 2 is troubling. The fact that this epidemic of hepatitis occurred during the SARS Cov 2 pandemic should of course be kept in mind. However the role of SARS Cov 2, of adenovirus and of their association as direct necrotic agent or as a trigger of inflammatory and/or immune reaction remains to be proven.^11^, ^12^ While some cases of acute hepatis have been linked to SARS Cov 2, the pathogenic role of SARS Cov2 is not proven. By the same way, the presence of adenovirus in infants is frequent and therefore, acute hepatitis can be totally independent of the presence of this virus. A first hypothesis was that a new subtype of adenovirus is the causing agent emerging, but there was not a specific subtype found in the reported cases. The second hypothesis is a synergistic role of the two viruses or an ability to trigger inflammatory destructive reaction within the liver. It has indeed been suggested that some cases of autoimmune hepatitis could be triggered by viral infection. In the Dutch cases as well as in the other cases, the histology was not in favour of autoimmune‐like hepatitis, nor viral hepatitis. Some authors have suggested a treatment with cidofovir in case of presence of adenovirus, the efficacy remains unproven.^13^

At this stage, there are more questions than answers. We must confirm the outbreak and understand the pathogenesis of acute liver failure and the respective role of viruses, immune and genetic factors in a such devastating disease.

## References

[1] Durand P , Debray D , Mandel R , Baujard C , Branchereau S , Gauthier F , et al. Acute liver failure in infancy: a 14‐year experience of a pediatric liver transplantation center. J Pediatr. 2001;139(6):871–6. 10.1067/mpd.2001.119989 11743517

[2] Bathia L , Grant WJ , Mercer DF , Vargas LM , Gebhart CL , Langnas AN . Parvovirus associated fulminant hepatic failure and aplastic anemia treated successfully with liver and bone marrow transplantation: a report of 2 cases. Am J Transplant. 2014;14:2645–50. 25179206 10.1111/ajt.12857

[3] Maggiore G , Socie G , Sciveres M , Roque‐Afonso AM , Nastasio S , Johanet C , et al. Seronegative autoimmune hepatitis in children: spectrum of disorders. Dig Liver Dis. 2016;48(7):785–91. 10.1016/j.dld.2016.03.015 27079745

[4] Coilly A , Dharancy S , Duvoux C , Cervoni JP , Dumortier J , Radenne S , et al. Prognosis of ALF of unknown cause: results of the French multicentre prospective HASIPRO study. Hepatology. 2018;68:192A–3A.

[5] Ichai P , Legeai C , Francoz C , Boudjema K , Boillot O , Ducerf C , et al. Patients with acute liver failure listed for super urgent liver transplantation: reevaluation of the Clichy Villejuif Criteria. Liver Transpl. 2015;21(4):512–23. 10.1002/lt.24092 25675946

[6] Marsh K , Tayler R , Pollock L , Roy K , Lakha F , Ho F , et al. Investigation into cases of hepatitis of unknown etiology among young children. Scotland 1 January 2022 to 12 April 2022. Euro Surveill. 2022;27(15):2200318. 10.2807/1560-7917.es.2022.27.15.2200318 PMC9012090 35426362

[7] Cates J , Baker JM , Almendares O , Kambhampati A , Burke RM , Belacharandran N , et al. Interim analyses of acute hepatitis of unknown etiology in children aged 10 years‐United States, October 2021‐June 2022. MMWR. 2022;71(26):852–8. 10.15585/mmwr.mm7126e1 35771734

[8] Kelgeri C , Couper M , Gupte GL , Brant A , Patel M , Johansen L , et al. Clinical spectrum of children with acute hepatitis of unknown cause. N Engl J Med. 2022;387(7):611–19. 35830627 10.1056/NEJMoa2206704

[9] Deep A , Grammatikopoulos T , Heaton N , Verma A , Dhawan A . Outbreak of hepatitis in Children: clinical course of children with acute liver failure admitted to the intensive care unit. Intensive Care Med. 2022;48(7):958–62. 10.1007/s00134-022-06765-3 35687162 PMC9184811

[10] Lexmond WS , De Meijer VE , Scheenstra R , Bontemps STH , Duiker EW , Scholvink EH , et al. Indeterminate pediatric acute failure: clinical characteristics of a temporal cluster of five children in the Netherlands in the Spring 2022. Unit Eur Gastroenterol J. 2022. 10.1002/ueg2.12269 PMC9557968 35773246

[11] Mucke MM , Zeuzem S . The recent outbreak of acute severe hepatitis in children of unknown origin: what is known so far. J Hepatol. 2022;77:237–42. 10.1016/j.jhep.2022.05.001 35533802

[12] Brodin P , Arditi M . Severe acute hepatitis in children: investigate SARS‐COV‐2 superantigens. Lancet Gastroenterol Hepatol. 2022; 7:594–5. 10.1016/S2468-1253(22)00166-2 PMC9106421 35576952

[13] Verma VS , Lampejo T , Deep A , Dhawan A . Use of cidofovir in recent outbreak of adenovirus‐associated acute liver failure in children. Lancet Gastroenterol Hepatol. 2022;7(8):700–2. 10.1016/s2468-1253(22)00199-6 35717991

